# Case report of subcutaneous panniculitis-like T-cell lymphoma complicated by eyelid swelling

**DOI:** 10.1186/s12886-016-0303-4

**Published:** 2016-07-20

**Authors:** Ryuya Hashimoto, Michihiro Uchiyama, Takatoshi Maeno

**Affiliations:** Department of Ophthalmology, Toho University Sakura Medical Center, 564-1 Shimoshizu, Sakura, 285-8741 Japan; Department of Hematology, Suwa Red Cross Hospital, 5-11-50, Suwa, 392-0027 Japan

**Keywords:** Subcutaneous panniculitis-like T-cell lymphoma, Eyelid swelling, Mucosal hyperemia, Ocular hypertension, Pancytopenia, Case report

## Abstract

**Background:**

Subcutaneous panniculitis-like T-cell lymphoma (SPTCL) is a rare, highly malignant, extranodal lymphoma that preferentially infiltrates into subcutaneous adipose tissue. No case of SPTCL with the earliest symptoms occurring in the eye region has been reported. We report a case of SPTCL complicated by eyelid swelling.

**Case presentation:**

A 19-year-old Japanese man presented with worsening left eyelid swelling. The patient’s best-corrected visual acuity (BCVA) was 0.8, left intraocular pressure was 36 mm Hg, and he had prominent mucosal hyperemia and edema. His left eye had light reflex attenuation and a positive relative afferent pupillary defect, but no abnormality in the visual field or central flicker value. Magnetic resonance imaging showed left orbital adipose tissue inflammation. The blood examination was normal. He was hospitalized for an intensive examination and treatment for possible cellulitis, orbital panniculitis, and inflammatory pseudotumor. Systemic antibiotics were initiated. The following day, he underwent a sub-Tenon’s injection of triamcinolone. Left eyelid swelling gradually improved. He was discharged on the ninth day and followed up with oral prednisolone. Two months later, he visited our department because of a high fever and slight right eyelid swelling. Ocular hypertension was detected. A blood examination revealed pancytopenia. Computed tomography showed fluid retention, hydrothorax, and abdominal dropsy. Magnetic resonance imaging revealed right orbital panniculitis. Because of suspected hemodyscrasia, he was referred to the hematology department of another hospital where he was diagnosed with SPTCL.

**Conclusions:**

The possibility of SPTCL, with attention to recurrence and systemic symptoms, should be considered in young patients with sudden eyelid swelling.

## Background

Subcutaneous panniculitis-like T-cell lymphoma (SPTCL) is a cytotoxic T-cell lymphoma that preferentially infiltrates into subcutaneous adipose tissue [1]. It is a rare disorder and represents less than 1 % of all non-Hodgkin lymphomas [2]. SPTCL is accompanied by fever, arthralgia, and hepatopathy as systemic symptoms, which cause hemophagocytic syndrome (HPS) in many patients [3].

In 1991, Gonzalez et al. [4] reported eight patients with similar clinicopathologies who exhibited a pathological mechanism of subcutaneous tissue-originated T-cell malignant lymphoma, which resulted in a poor prognosis after being complicated by HPS. In 1994, SPTCL was proposed as a provisional subtype of peripheral T-cell lymphoma under the Revised European American Lymphoma (REAL) classification [5]. In 2001, a new World Health Organization (WHO) classification established SPTCL as an independent disease [6]. Subcutaneous nodules focused on the extremities and trunk are frequently the initial symptoms of SPTCL, and they frequently accompany systemic symptoms [3]. Among the malignant lymphomas, the initial symptoms of SPTCL tend to appear in relatively young patients, with a mean age of onset of 33 years [7]. No standard treatment for SPTCL has been established. However, various treatments such as chemotherapy, radiotherapy, bone marrow transplantation, and immunosuppressive therapy have been reported [1, 2].

To our knowledge, no case of SPTCL with the earliest symptoms presenting in the eye region has been reported. In this paper, we report a case of SPTCL complicated by swelling in one eyelid, which was the original symptom. The eyelid swelling temporarily improved with treatment. Approximately 3 months after the initial treatment, systemic symptoms and SPTCL reappeared, complicated by swelling of the opposite eyelid.

## Case presentation

**Fig. 1:**
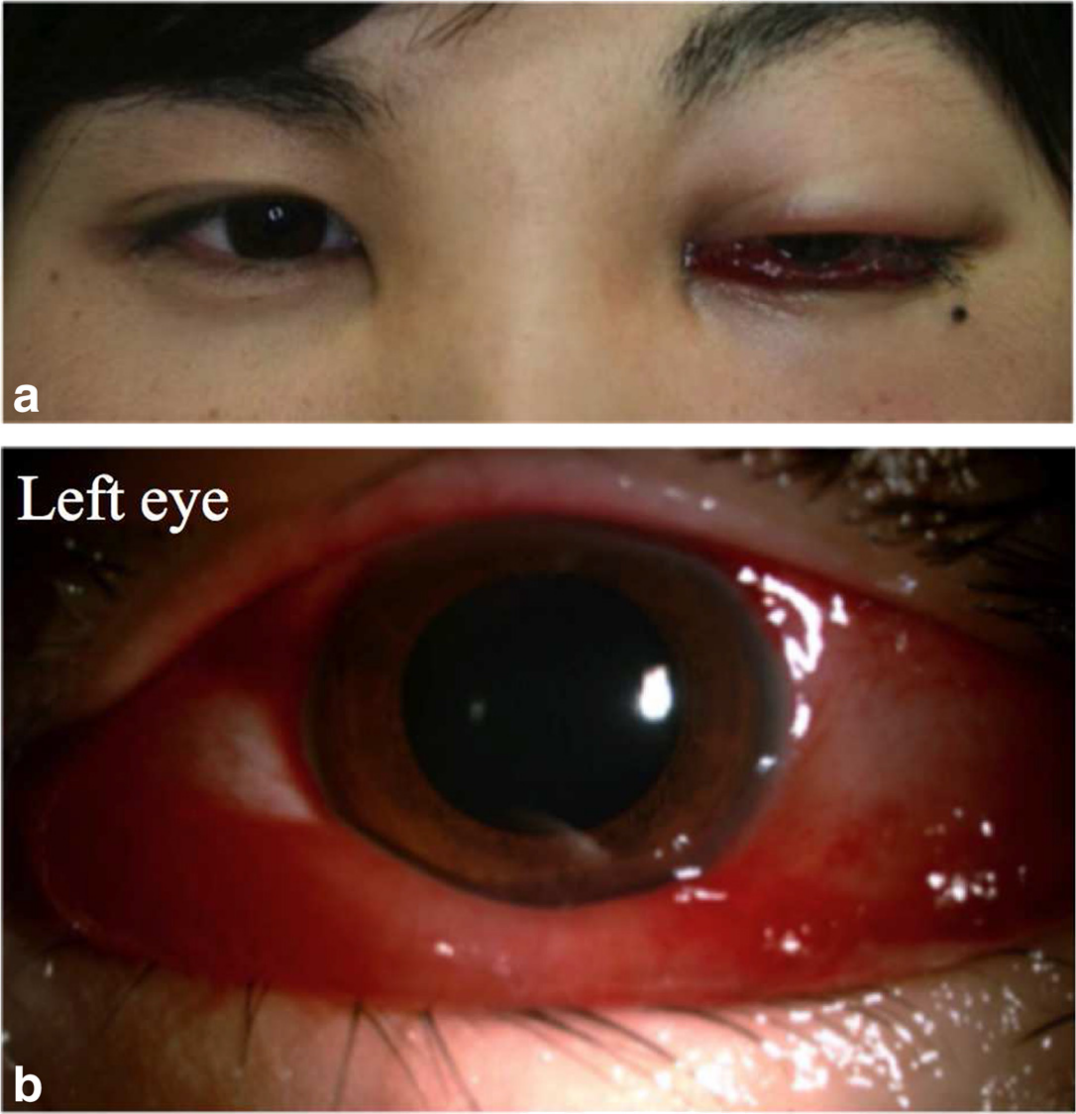
The patient’s face and left anterior ocular segment at the first visit. **a** The patient exhibits prominent left eyelid swelling and exophthalmos. **b** A photograph demonstrates prominent mucosal hyperemia, edema, and moderate mydriasis of the pupil

A 19-year-old Japanese man developed left eyelid swelling around October 2, 2013. On October 9, 2013, he visited the ophthalmologic department of a local hospital because of subsequent gradually worsening ophthalmalgia and visual deterioration. The symptoms did not improve after the ocular administration of antibiotics. He was referred to Toho University Sakura Medical Center in Sakura, Japan (referred to hereafter as “our hospital”) on October 16, 2013, with a main complaint of left eyelid swelling. He had no medical, family medical, or allergy history. At the first visit, his right best-corrected visual acuity (BCVA) was 1.2; his left BCVA was 0.8 with visual deterioration. His right and left intraocular pressures (IOPs) were 17 mmHg and 36 mmHg, respectively. Prominent left eyelid swelling and exophthalmos were evident (Fig. 1a). A slit-lamp examination revealed prominent mucosal hyperemia, edema, and slight mucous discharge (Fig. 1b). Moderate mydriasis of the left pupil, direct light reflex attenuation, and positive relative afferent pupillary defect (RAPD) were present. No abnormality existed in the corneas, lenses, retinas, or optic nerve heads; however, ocular motility was omnidirectionally circumscribed. Dynamic campimetry showed neither central scotoma nor visual field deficiency. No laterality of central flicker values was revealed on central flicker examination. No systemic symptoms such as fever or infection were found on physical examination at the first visit. The blood examination exhibited a normal inflammatory response value.

**Fig. 2:**
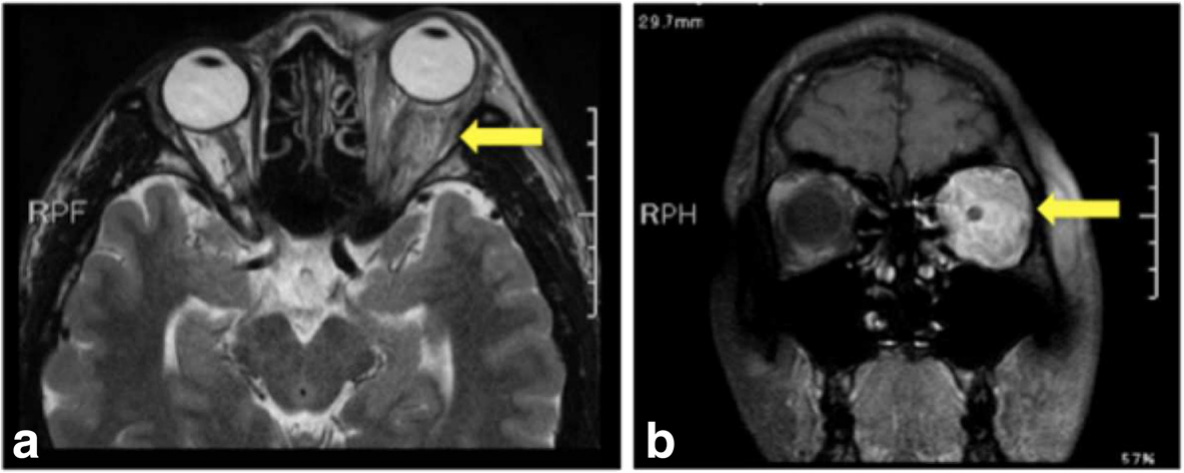
Orbitographic magnetic resonance imaging and a T_2_-weighted image from the first visit. **a** A systematic trachychromatic image of the left periorbital soft tissues and (**b**) soft tissue in the left orbital muscle cone is swollen, and the left eyeball is protruding

Orbital magnetic resonance imaging (MRI) showed soft tissue swelling in the left orbital muscle cone, and orbitographic MRI showed systematic trachychromatic images in the periorbital soft tissues (Fig. 2).

Clinical findings indicated suspected orbital cellulitis, although the blood examination exhibited no increase in the inflammatory response value. Intraorbital inflammatory pseudotumor was a differential diagnosis because of suspected orbital panniculitis on diagnostic MRI. He was hospitalized. An intravenous infusion of betamethasone (4 mg/day) was started on October 16, 2013.

**Fig. 3:**
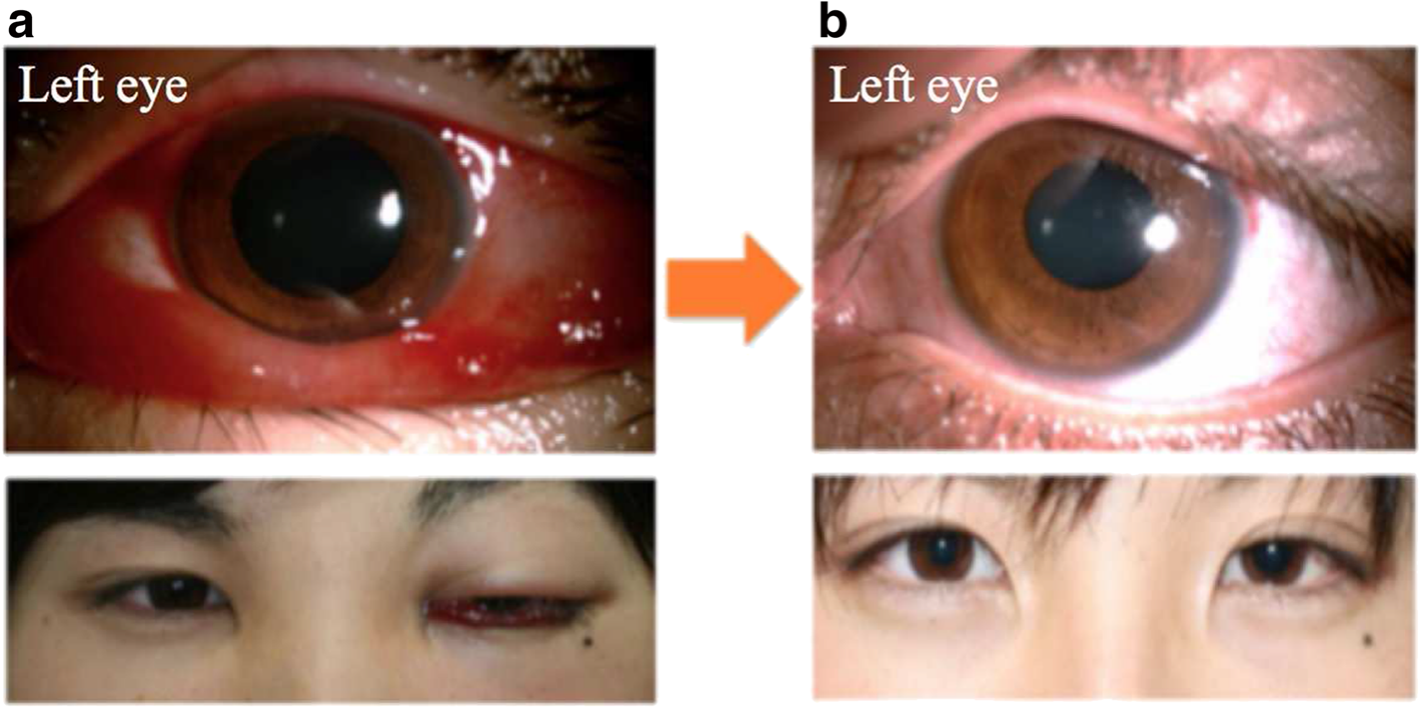
The anterior ocular segment before and after treatment. Images of the patient’s eyes (**a**) before treatment (October 16, 2013) and (**b**) after treatment (January 4, 2014) are shown. The mucosal hyperemia and edema improved, and the eye swelling disappeared by the third month after starting treatment

The following medications were started at the first visit because of the possibility of orbital cellulitis: ocular administration of levofloxacin four times daily and cefmenoxime four times daily, ofloxacin ophthalmic ointment once daily, and intravenous infusions of imipenem/cilastatin (0.5 g × 3 times daily). Culture identification of the eye discharge was also performed on the same day. Oral administration of acetazolamide tablets (300 mg × 3 × 3 times daily) and potassium l-aspartate tablets (300 mg × 3 × 3 times daily) was also started because of left ocular hypertension. We supposed that there might be another possibility of an immunologic or inflammatory pseudo tumor besides orbital cellulitis because of the few inflammatory clinical findings; thus, we tried to use a sub-Tenon’s injection of triamcinolone as a diagnostic therapy. A sub-Tenon’s injection of triamcinolone (0.5 mg) in the left eye on the first day of hospitalization resulted in improvements in mucosal hyperemia and edema and the disappearance of eyelid swelling (Fig. 3). The left BCVA improved to 1.2, there was a reduction in left IOP to 15 mmHg, and the left eyelid swelling nearly disappeared and did not recur. Mucosal hyperemia and edema were observed on the sixth day of hospitalization. Improvement of the left orbital panniculitis compared to the image acquired at the first visit was revealed by orbitographic MRI on the seventh day of hospitalization. He was discharged on the ninth day of hospitalization. Left and right IOPs gradually declined to approximately 15 mm Hg at discharged. Systemic and local administrations of antibiotics were discontinued on the seventh day of hospitalization because a culture of the eye discharge, which was performed at the first visit, had returned negative results. Oral prednisolone (30 mg/day) was started after hospital discharge. There was no recurrence of ocular symptoms and a favorable progression in vision to 1.2, despite a gradual reduction of prednisolone. Favorable progress in the left ocular tension to approximately 15 mmHg also occurred with the discontinuation of oral acetazolamide tablets and the initiation of the ocular administration of dorzolamide hydrochloride/timolol maleate (twice) and latanoprost (once).

Symptoms had not recurred when he was examined at an ambulatory follow-up visit on January 8, 2014. On January 20, 2014, he visited the internal medicine department of a local hospital and was orally administered antibiotics because of a subsequent fever of 39 °C and appendicular arthralgia. The symptoms did not improve, despite the oral antibiotic administration. A subsequent blood examination exhibited pancytopenia, hepatopathy, and an increased lactate dehydrogenase (LDH) level. He immediately revisited our department for an intensive examination and treatment on January 29, 2014 (the 15^th^ week after the first visit) with a complaint of slight swelling of the right eyelid, whereas swelling of the left eyelid was observed at the first visit.

**Fig. 4:**
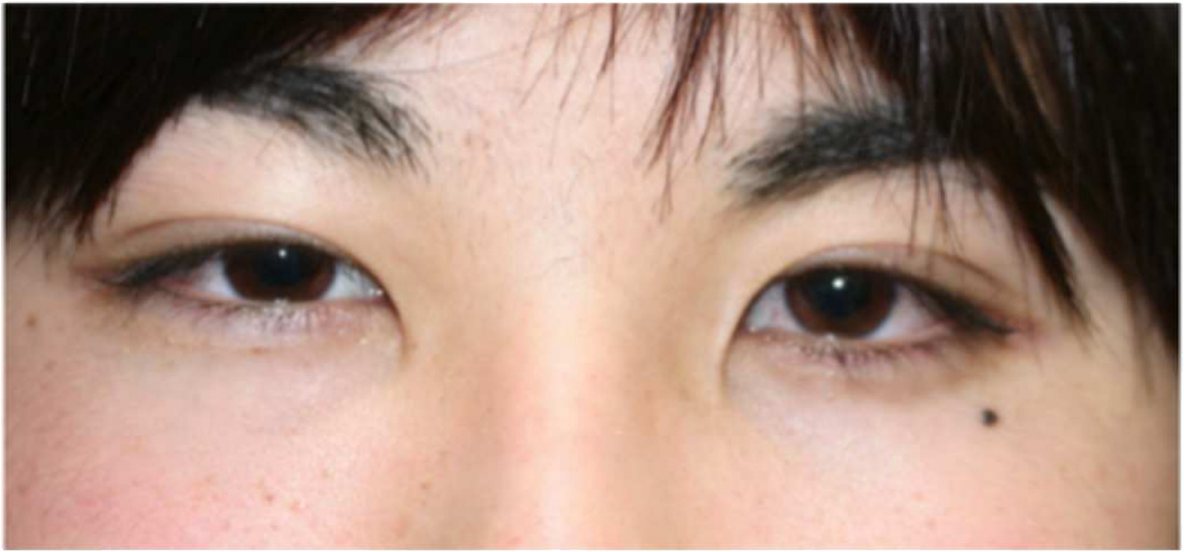
The patient’s face at recurrence. The patient shows slight right eyelid swelling (i.e., the opposite eyelid)

**Fig. 5:**
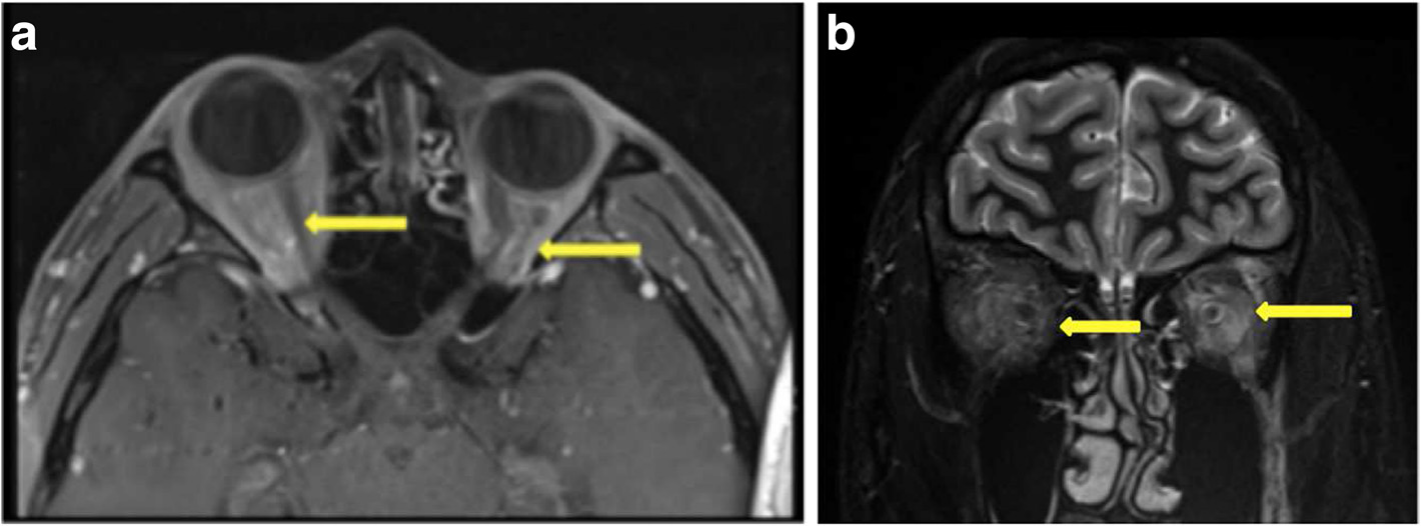
Orbitographic magnetic resonance imaging at recurrence. Right exophthalmos (a) and orbital softtissue inflammation (b) are evident

Ophthalmological findings at the second visit revealed slight right eyelid swelling (Fig. 4). The BCVA of both eyes was 1.2, but the intraocular pressure was 25 mmHg in the right eye and 27 mmHg in the left eye. Slight direct light reflex attenuation of both eyes was present, and positive RAPD of the right eye was also found. No abnormality was found in dynamic campimetry or a central flicker examination. However, orbitographic MRI showed right exophthalmos and orbital soft tissue inflammation. Left orbital panniculitis, which had improved (compared to the image acquired at the first visit), had recurred, compared to the image acquired during hospitalization on October 23, 2013 (Fig. 5). Systemic findings included a high body temperature of 39.8 °C and tachycardia of 103 beats per minute. A blood examination revealed leucocyte and thrombocyte cytopenia, hepatopathy, and increased coagulability: leucocytes, 3220/mm^3^; thrombocytes, 11.1 × 104 mm^3^; aspartate transaminase/alanine transaminase (AST/ALT) level, 115/91 IU/L; LDH level, 1014 IU/L; creatinine kinase level, 362 IU/L; prothrombin time, 13.6 s; activated partial thromboplastin time (APTT), 37 s; and D-D dimer, 2.17 s. He visited the internal medicine department of our hospital for an intensive examination. Abdominal echography and abdominal/pelvic computed tomography (CT) showed moderate retention of abdominal fluid, dropsy, and an enlarged spleen.

He was transferred to the hematology department at another hospital and was hospitalized for an intensive examination on the following day because of the possibility of hemodyscrasia, based on the increased LDH level in the blood examination results. Bone marrow aspiration examined after the hospital transfer revealed decreased numbers of nucleated cells and megakaryocytes and a small amount of corpuscular phagocytosis. A blood examination revealed leucocyte and thrombocyte cytopenia; increased levels of AST/ALT, LDH, and ferritin; and hepatopathy. Antibodies against herpes simplex virus, cytomegalovirus, and Epstein–Barr virus were negative.

**Fig. 6:**
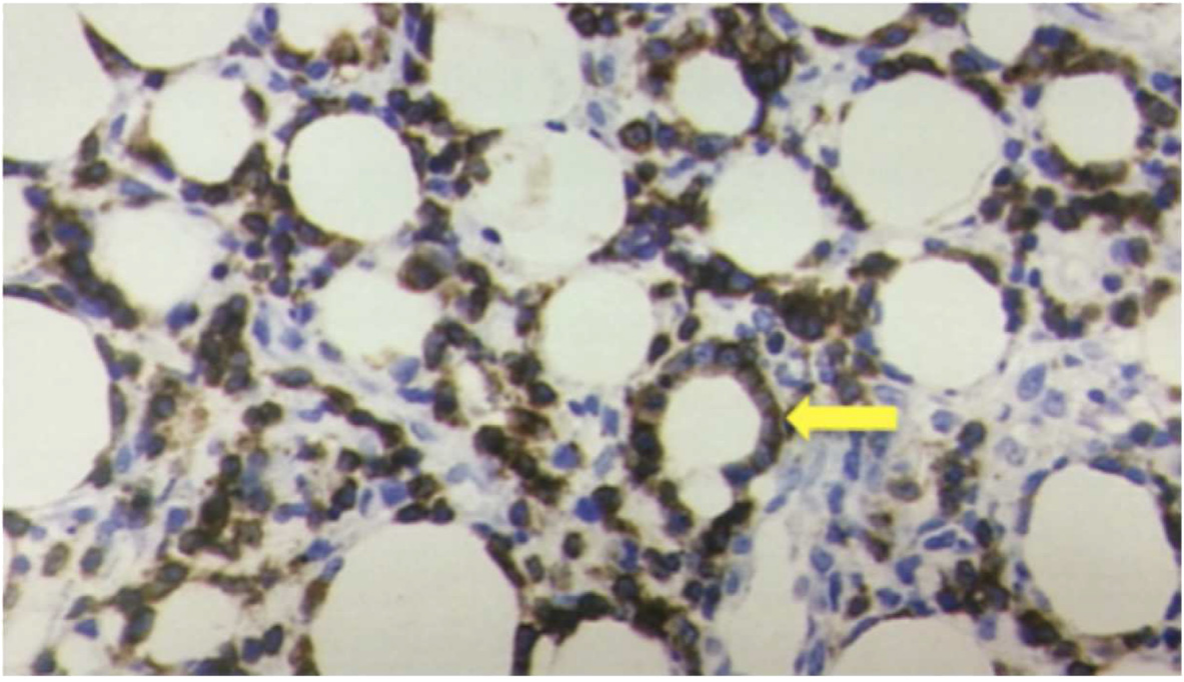
The histopathological findings of the vesicular subcutaneous tissue. The immunohistochemistry shows that the infiltrating lymphocytes are positive for CD8. There is an aggregating tendency of atypical lymphocytes and a surrounding arrangement (i.e., rimming image) of lipid droplets (CD8, magnification, 400×)

Steroid administration (30 mg/day) was started with a diagnosis of HPS. After 1 week, he left the hospital because his symptoms improved. However, bubonalgia appeared immediately after hospital discharge and did not improve. He revisited the hospital on February 29, 2014. Abdominal CT exhibited suspected panniculitis in vesicular subcutaneous tissue. Cyclosporine was administered because of suspected Weber–Christian disease. The hepatopathy and increased levels of LDH and ferritin did not improve. Worsening HPS was diagnosed because of the appearance of a high fever (39.5 °C) on the morning of March 20, 2014. A biopsy of the vesicular ventral subcutaneous adipose tissue by hypogastric transection was also performed in the anaplastic department of a local hospital on the same day. The histopathological diagnosis of the biopsy tissue revealed an aggregating tendency of nuclear irregularity-prominent atypical lymphocytes in the adipose tissue. SPTCL was diagnosed because of the surrounding arrangement (i.e., rimming pattern) of lipid droplets (Fig. 6). On May 1, 2014, two aggregations of fluorodeoxyglucose in the right ilium were detected by positron emission tomography (PET) examination. Advanced malignant lymphoma was also suspected, based on the pelvic MRI findings. He was transferred to Suwa Red Cross Hospital (Suwa, Japan) on May 1, 2014 because of his requirements for intensive examination and treatment.

After the hospital transfer, histopathological and immunohistological re-examinations of the vesicular adipose tissue exhibited poor infiltration of atypical lymphocytes into the dermis. Immunohistological findings exhibited positivity for CD3, CD8, granzyme B, T-cell intracellular antigen-1 (TIA-1), and beta F1; and negativity for C-gamma M1, CD30, CD56, and Epstein–Barr virus-encoded small RNAs (EBERs), which led to a definitive diagnosis of αβ-type SPTCL. He was hospitalized for treatment on May 21, 2014. The standard treatment for malignant lymphomas—cyclophosphamide, doxorubicin, vincristine, and prednisone (CHOP) therapy—was administered for 5 days, and presented no efficacy. Four courses of etoposide, methylprednisolone sodium succinate, high-dose cytarabine, and platinum chemotherapy were administered beginning on June 11, 2014. On September 26, 2014, he underwent autologous peripheral stem cell transplantation because his symptoms were controlled. Hospital treatment for 6 months resulted in remission of his symptoms. Remission has been maintained.

## Conclusions

The patient originally presented with swelling of one eyelid complicated by visual deterioration. Systemic symptoms such as high fever, abdominal dropsy, and hepatosplenomegaly subsequently appeared after the temporary improvement of symptoms by local/systemic steroid administration. This improvement was followed by swelling of the right (i.e., the opposite) eyelid. The findings at the first visit were similar to findings of orbital cellulitis. No fever and no increase in the inflammatory response value were indicated by the blood examination. Previous cases of SPTCL have been reported with many patients originally presenting with subcutaneous nodules on the trunk and/or extremities, and systemic symptoms such as fever and arthralgia [3]. No case of opposite eyelid swelling with systemic symptoms occurring after temporary symptom improvement without systemic involvement in the early stages of SPTCL has been reported previously.

This case of SPTCL was very difficult to differentiate. The appearance of facial symptoms is rare; based on 83 recently reported cases, the original appearance of symptoms was on the face in only 16 cases, compared to the lower extremities in 64 cases, the upper extremities in 52 cases, and the trunk in 47 cases [7].

SPTCL is difficult for doctors in other departments to differentiate, and frequently leads to a first diagnosis of cellulitis or subcutaneous panniculitis. Symptoms of eyelid swelling occur in orbital phlegmon, orbital inflammatory pseudotumor, immunoglobulin G_4_ (IgG_4_)-related diseases, malignant orbital lymphomas, and collagenosis such as scleroderma and systemic lupus erythematosus.

Orbital cellulitis was excluded from the differential diagnosis during the treatment process, based on clinical findings such as the absence of fever and lymphoma at the first visit, the absence of an increase in inflammatory response value in the blood examination, and no detection of pathogenic bacteria in the culture identification of the eye discharge. Furthermore, collagenosis and IgG_4_-related diseases were excluded because the blood examination detected no autoantibody accompanying collagenosis and because of the normal IgG value.

Malignant orbital lymphomas, an important differential disease, frequently appear with eyelid swelling as the original symptom. Most orbital malignant lymphomas accompany B-cell lymphomas, which exhibit relatively good prognoses compared with prognoses of malignant lymphomas originating in other regions. Orbital malignant lymphomas often occur in young adults; however, the onset is usually in old age [8–10]. MRI examination demonstrates the boundaries of tumors more clearly in malignant lymphomas than in orbital inflammatory pseudotumors [11, 12], although a pathological examination is required for a definitive diagnosis of malignant lymphoma.

Progress observation instead of a highly invasive biopsy of the orbital soft tissue was performed in this patient after consultation with neurosurgeons at our hospital because of the rapid improvement of symptoms after the initial and steroid treatments and the lack of recurrence. Orbital malignant lymphoma was excluded because of the young age of onset (19 years), the early remission of symptoms after steroid administration, and the uniform staining of soft tissue indicated by MRI. Therefore, the initial treatment for the most suspected disease—orbital panniculitis—was started. It is necessary to attempt to detect diseases early by biopsy, gallium scintigraphy, and PET examinations (depending on progress observation) because of the possibility of orbital malignant lymphoma and metastases from other organs.

SPTCL is divided into the αβ-type and the γδ-type, based on histopathological and immunohistological findings. The prognoses differ between these two types. Because complications occur more frequently in γδ-type SPTCLs with HPS than in αβ-type SPTCLs during the clinical course, the prognoses of αβ-type SPTCLs are relatively better than the prognoses of γδ-type SPTCLs, which are complicated frequently by HPS [7]. Massone et al. [13] reported that the 5-year survival rate of αβ-type SPTCL was approximately 80 %. On the other hand, investigators of a European multicenter study [7] reported that the 5-year survival rates among 63 cases of αβ-type SPTCL were 46 % for HPS-complicated cases (11 cases) and 91 % for cases not complicated with HPS (52 cases), and that the 10-year survival rates were equivalent to the 5-year survival rates.

No standard treatment for SPTCLs has been established. However, various treatments such as chemotherapy, radiotherapy, bone marrow transplantation, and immunosuppressive therapy have been reported, and patients frequently respond to oral steroid administration in the early stage of the disease and easily respond to combination chemotherapy [1, 2, 14].

With regard to the possibility of symptom relapse after the reduction of steroid treatment, the reappearance of systemic symptoms after the discontinuation of oral steroid treatment after temporary symptom remission resulting from betamethasone infusion and oral steroid administration is consistent with the progression of SPTCL in this patient. The reasons for the improvement in eyelid margin swelling with steroid administration in the early stage of the disease may have been related to responses to anti-inflammatory and antitumor actions. No standard treatment for SPTCLs has been established, although the effectiveness of peripheral stem cell transplantation within 1 year of onset has been reported. When symptoms appear and/or worsen during treatment, an early and accurate histopathological diagnosis by biopsy in the early stage should be performed, and peripheral stem cell transplantation should be considered as an alternative.

SPTCL is a relatively novel concept. Possible cases of SPTCLs previously diagnosed as Weber–Christian disease and HPS cannot be denied. Because there are fewer cytotoxic T-cells with positivity for CD8 in the initial SPTCL lesion, a re-examination of biopsy tissue often exhibits a characteristic picture and histology of SPTCL after the diagnosis of Weber–Christian disease, lupus profundus, or HPS by a histopathological diagnosis in the early stage of the disease. Therefore, when SPTCL is suspected, and even when SPTCL is excluded by the initial biopsy, the necessity of biopsy re-examination without hesitation should be kept in mind. SPTCL, which is even likely to appear in young adults, is a very rare disease. Delays in diagnosis and/or treatment may influence the vital prognosis.

This rare case of SPTCL originally presented with eyelid symptoms, no fever, and no systemic symptoms, such as hepatosplenomegaly. This case subsequently exhibited systemic symptoms and swelling of the opposite eyelid after a certain period of time. When rapidly worsening eyelid swelling occurs in relatively young adults, the possibility of hemodyscrasia such as SPTCL should be considered. Furthermore, the appearance of systemic symptoms and the recurrence of eyelid swelling should be considered during progress observation.

## Abbreviations

APTT, activated partial thromboplastin time; AST/ALT, aspartate transaminase/alanine transaminase; BCVA, best-corrected visual acuity; CHOP, cyclophosphamide, doxorubicin, vincristine, and prednisone; CT, computed tomography; EBERs, Epstein–Barr virus-encoded small RNAs; IOP, intraocular pressure; LDH, lactate dehydrogenase; MRI, magnetic resonance imaging; PET, positron emission tomography; RAPD, relative afferent pupillary defect; REAL, Revised European American Lymphoma; SPTCL, subcutaneous panniculitis-like T-cell lymphoma; TIA-1, T-cell intracellular antigen-1; WHO, World Health Organization
